# Acute Isolated Volar Distal Radioulnar Joint Dislocation Treated With Closed Reduction and Percutaneous Kirschner Wire Fixation: A Case Report

**DOI:** 10.7759/cureus.114749

**Published:** 2026-08-18

**Authors:** Yuri De Freitas Tobias, André Figueiredo Bordini, João Vitor Cardoso Moraes, João Paulo Rodrigues Pacheco

**Affiliations:** 1 Department of Orthopedics and Traumatology, Santa Casa de Misericórdia de São Sebastião do Paraíso, São Sebastião do Paraíso, BRA

**Keywords:** a case report, distal radioulnar joint (druj), druj, kirschner wire (k-wire), volar dislocation, wrist

## Abstract

Isolated volar dislocation of the distal radioulnar joint (DRUJ) without associated fracture is a rare wrist injury that may be overlooked because clinical and radiographic findings can be subtle. We report the case of a 19-year-old male rodeo rider who presented with severe left wrist pain, swelling, and inability to rotate the forearm following a torsional injury. Radiographs demonstrated an isolated volar DRUJ dislocation without associated fracture. Initial closed reduction was unsuccessful. Under anesthesia, reduction was achieved but was unstable, requiring percutaneous radioulnar transfixation with Kirschner wires and immobilization. At the two-month follow-up, the patient was pain-free, had preserved pronation and supination, and demonstrated a clinically stable DRUJ. This case highlights the importance of early recognition, appropriate reduction, and stabilization when instability persists after reduction.

## Introduction

Distal radioulnar joint (DRUJ) dislocations are most commonly associated with fractures of the radius and ulna. Isolated dislocation of this joint is uncommon. When DRUJ dislocation is isolated, dorsal displacement is more common, whereas volar displacement is rare. The DRUJ is a diarthrodial pivot joint that allows forearm pronation and supination through coordinated rotation and translation of the radius around the relatively fixed ulna. Its stability depends on both osseous congruity and soft-tissue structures, particularly the triangular fibrocartilage complex (TFCC), which includes the dorsal and palmar radioulnar ligaments. The palmar radioulnar ligament becomes taut during pronation, whereas the dorsal radioulnar ligament becomes taut during supination. Disruption of these stabilizing structures can result in DRUJ instability and, in rare cases, isolated volar or dorsal dislocation [1,2].

The attending physician should be aware of the clinical signs, which may be subtle, as well as the radiological findings, which can be difficult to interpret. Early diagnosis and prompt management are essential to achieve favorable outcomes for the patient [3].

## Case presentation

A 19-year-old male rodeo rider presented to the emergency department with severe left wrist pain of one day’s duration. The pain was associated with local swelling and an inability to pronate the forearm. During a ride, he was unable to release his left hand from the support on the animal, resulting in a severe twisting injury to the wrist.

Physical examination revealed swelling and tenderness around the wrist. The forearm was fixed in hypersupination of the DRUJ, with a prominent and easily palpable ulnar head, as well as marked limitation of forearm rotation due to pain.

**Figure 1:**
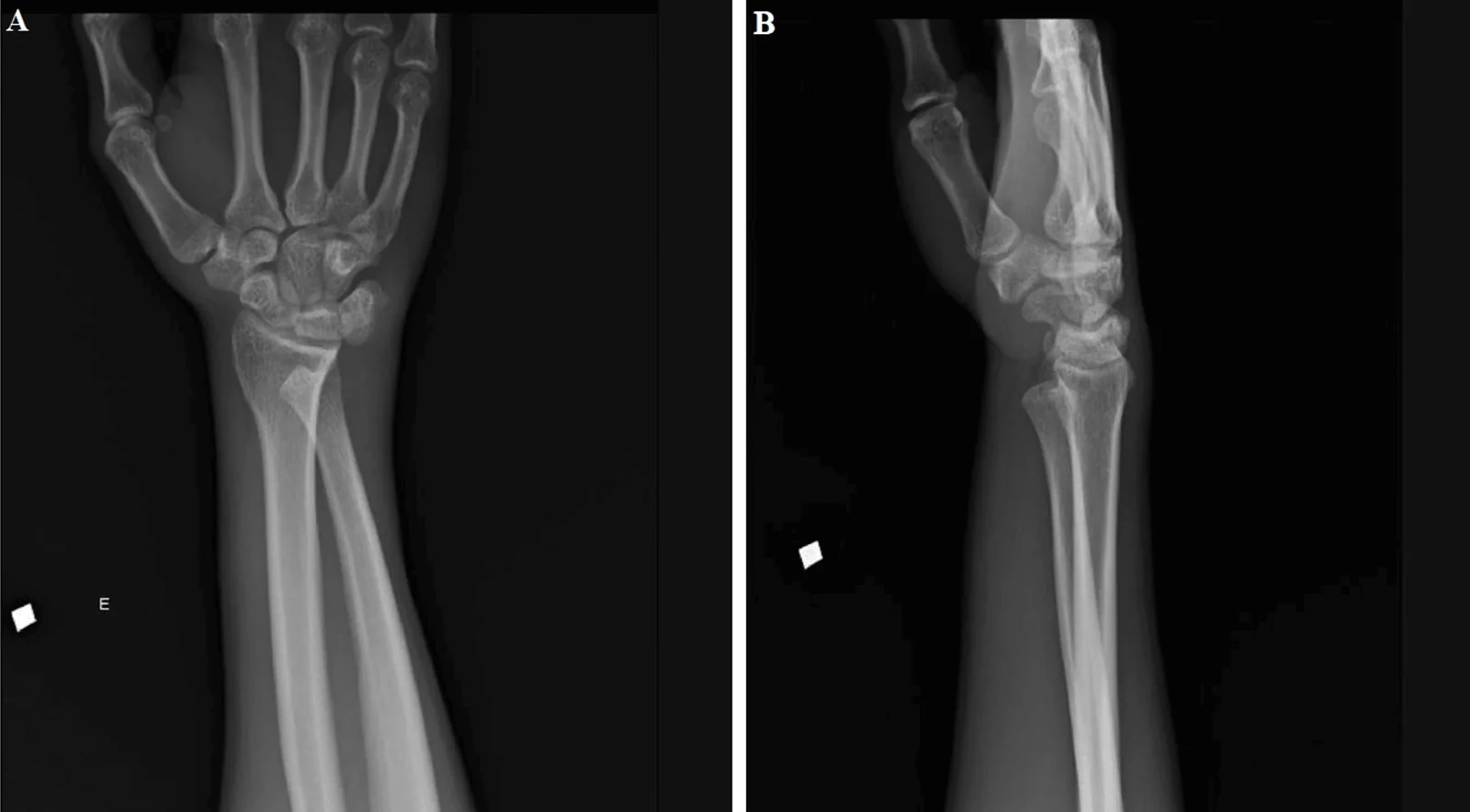
Initial radiographs of the left wrist. (A) Anteroposterior view. (B) Lateral view demonstrating isolated volar dislocation of the DRUJ without associated fracture. DRUJ: distal radioulnar joint

No signs of neurovascular compromise were identified. Plain radiographs of the left wrist in anteroposterior and lateral views demonstrated an isolated volar dislocation of the DRUJ without an associated fracture of the radius or ulna (Figure 1).

Initially, an attempt at closed reduction of the joint was made in the emergency department. Following the reduction maneuver, the joint was easily manipulated; however, the reduction was unstable, and the joint immediately returned to its dislocated position. Consequently, surgical treatment was scheduled for the following day.

**Figure 2:**
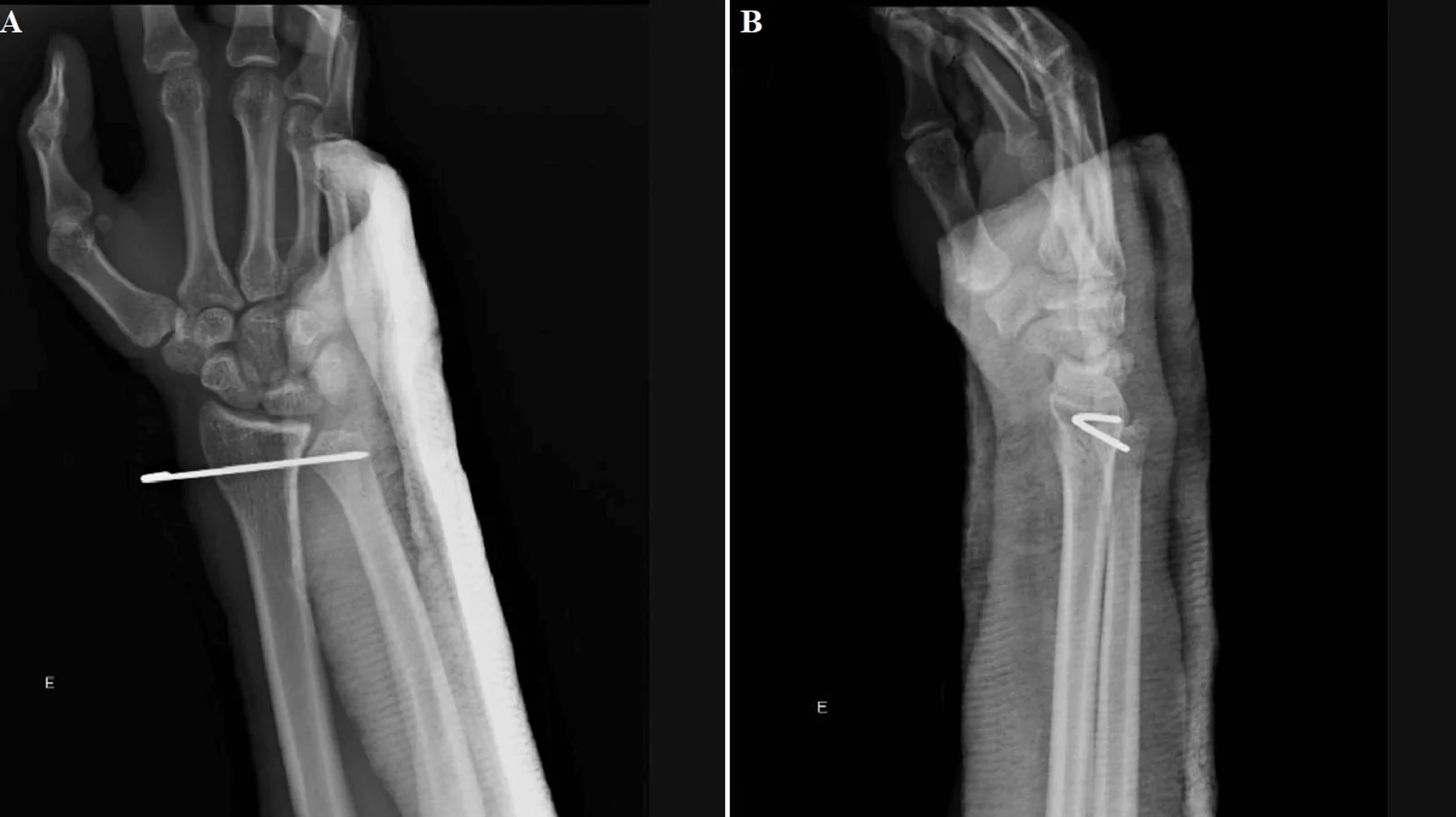
Immediate postoperative radiographs after closed reduction and percutaneous radioulnar transfixation with Kirschner wires. (A) Anteroposterior view. (B) Lateral view.

In the operating room, closed reduction of the DRUJ was performed under anesthesia, with satisfactory restoration of anatomical alignment. As instability persisted after reduction, percutaneous radioulnar transfixation was performed using 2.0-mm Kirschner wires, followed by immobilization with a forearm plaster splint (Figure 2).

**Figure 3:**
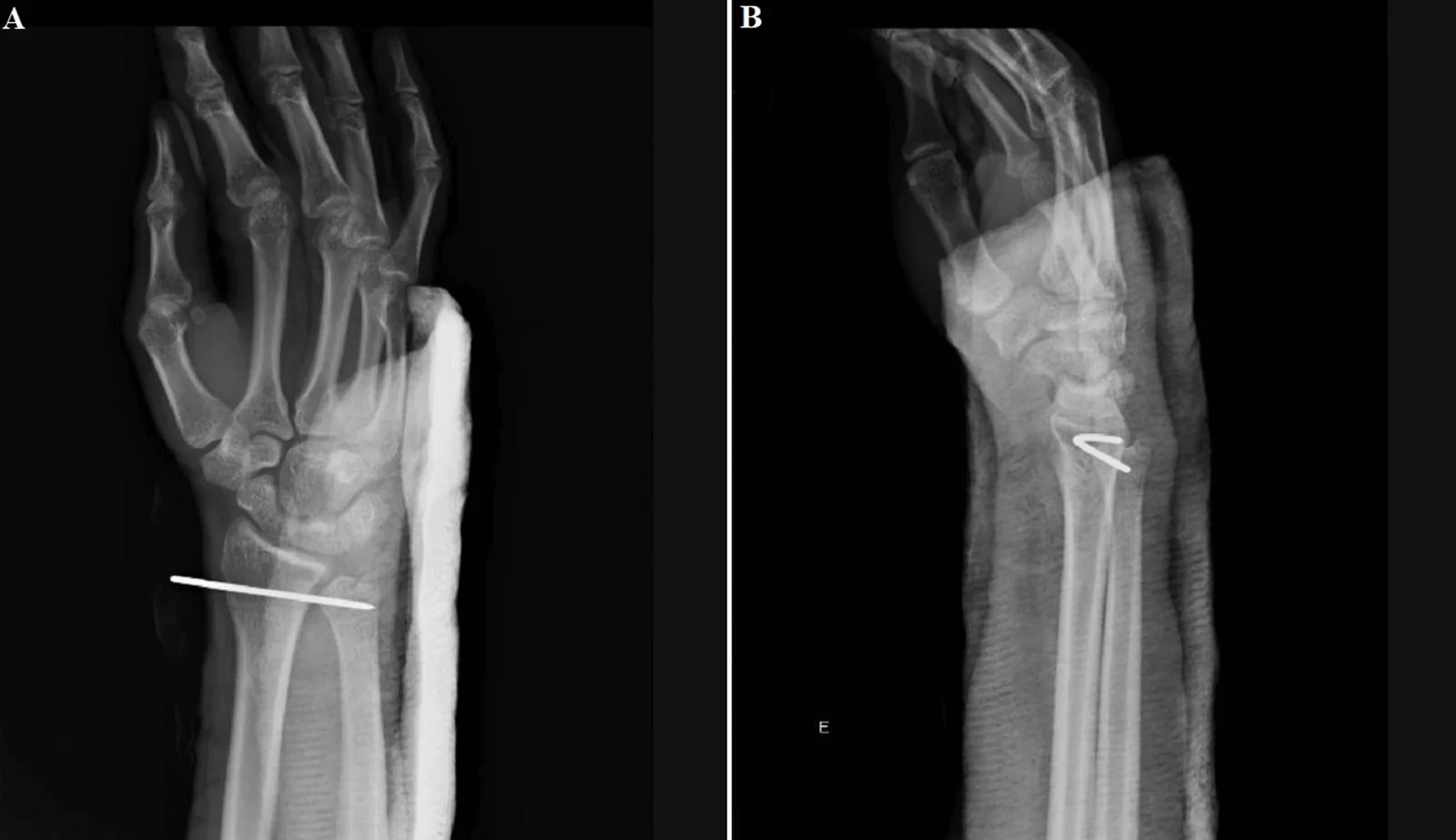
Follow-up radiographs 20 days after surgery showing maintained DRUJ reduction with percutaneous fixation and immobilization. (A) Anteroposterior view. (B) Lateral view. DRUJ: distal radioulnar joint

**Figure 4:**
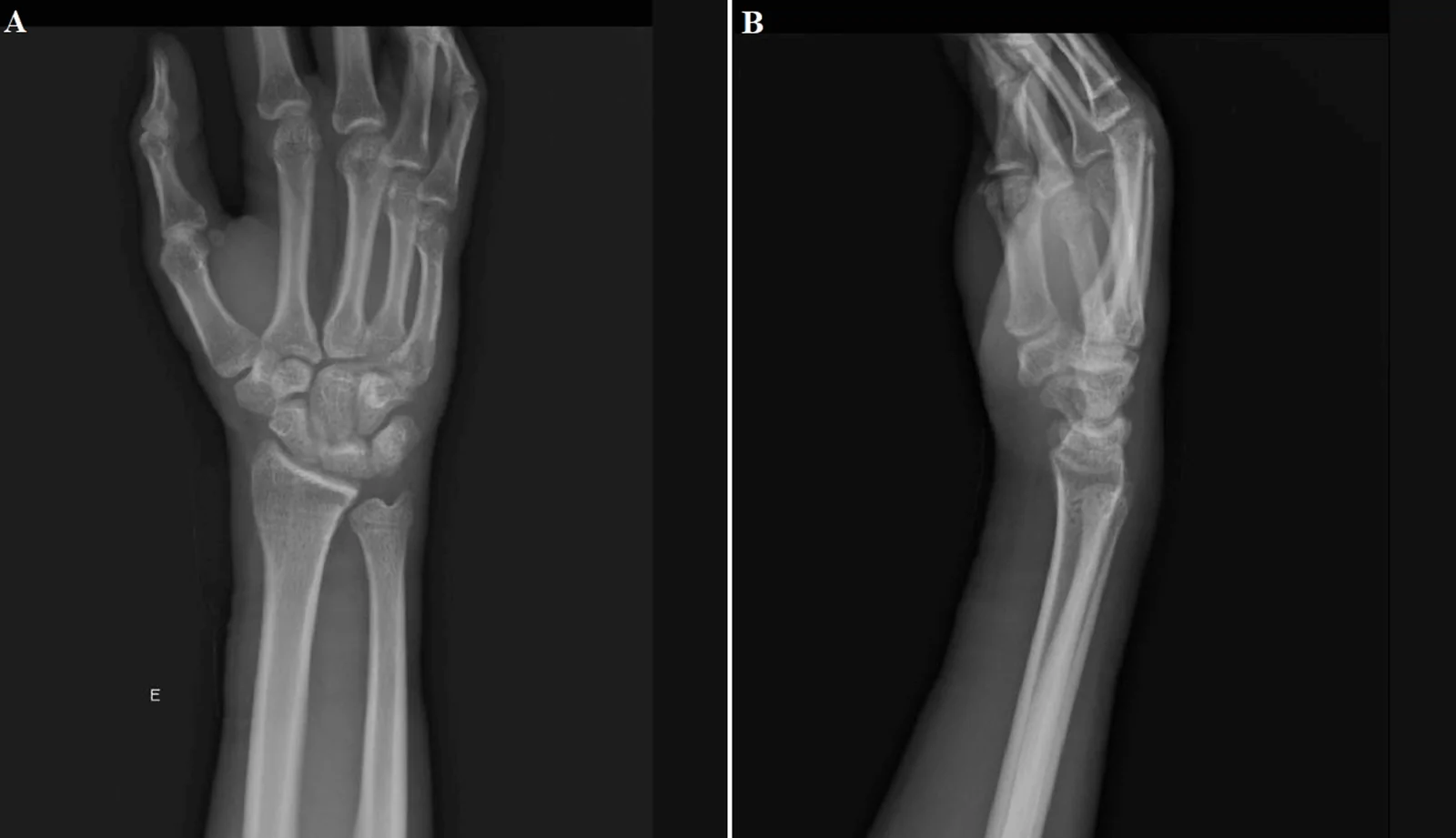
Radiographs four weeks after surgery following removal of the Kirschner wires and plaster splint. (A) Anteroposterior view. (B) Lateral view showing maintained alignment.

**Figure 5:**
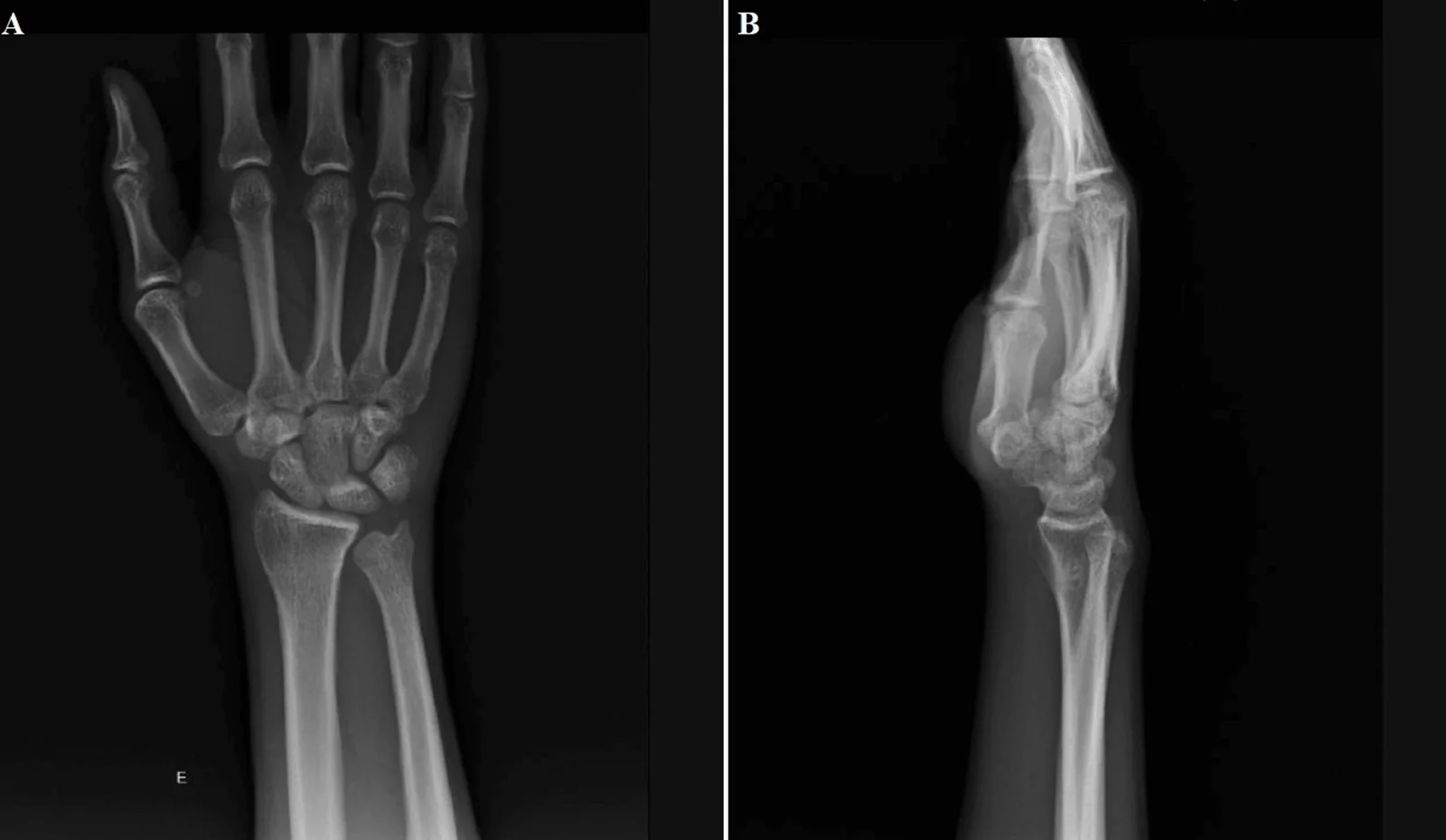
Radiographs at two-month follow-up. (A) Anteroposterior view. (B) Lateral view showing maintained DRUJ alignment after removal of fixation. DRUJ: distal radioulnar joint

The patient was followed weekly in the outpatient clinic. No wound complications, neurovascular symptoms, or recurrent dislocation were observed. Follow-up radiographs obtained 20 days after the procedure demonstrated maintenance of the reduction (Figure 3). Four weeks after surgery, the splint and Kirschner wires were removed (Figure 4). At two months postoperatively, the patient was pain-free and had preserved pronation and supination, with a clinically stable DRUJ (Figure 5).

After the initial two-month follow-up period, the patient did not attend subsequent scheduled appointments and was therefore lost to long-term follow-up. CT and MRI were not performed due to resource limitations at the healthcare facility.

## Discussion

The DRUJ is a diarthrodial pivot joint that allows rotation and translation of the radius around the relatively fixed distal ulna. The rounded head of the ulna articulates with the sigmoid notch located on the medial aspect of the distal radius. The TFCC serves as the primary stabilizer of the joint and contributes to the connection between the radius and ulna in this region. This structure is interposed between the distal end of the ulna and the most medial portion of the carpal bones, particularly the triquetrum. It originates from the ulnar notch of the radius and inserts into the base of the ulnar styloid process. The TFCC measures approximately 1 to 2 mm in thickness, has peripheral vascularization, and is composed of two peripheral ligaments: the dorsal radioulnar ligament, which extends from the dorsal aspect of the radius to the ulnar fovea and becomes tense during supination, and the palmar radioulnar ligament, which extends from the anterior aspect of the radius and also inserts into the ulnar fovea, becoming tense during pronation. Centrally, there is a cartilaginous disc that blends with the articular cartilage of the radius. The TFCC is also responsible for separating the cavities of the DRUJ and the radiocarpal joint [3-5].

Because isolated DRUJ dislocation is rare and DRUJ dislocation is usually associated with fracture, most published cases continue to be reported as individual case reports, even decades after the earliest descriptions. Few reviews, systematic studies, or other robust investigations have been conducted. Consequently, there remains a lack of consensus regarding the pathophysiology, optimal diagnostic imaging modality, and definitive treatment of this injury. The most widely accepted traumatic mechanism involves hypersupination of the forearm in cases of volar dislocation and hyperpronation in cases of dorsal dislocation [6,7].

The diagnosis may be missed during the initial emergency evaluation in up to 50% of cases, as the radiographic pattern is difficult to interpret and is rarely encountered in daily practice. Additionally, proper patient positioning for adequate radiographic views may be challenging due to pain [1,6]. Several authors recommend obtaining radiographs of the contralateral wrist in a rotational position comparable to that of the affected wrist, allowing comparison between the two wrists. In the present case, however, contralateral wrist radiographs were not obtained.

CT without contrast is useful for assessing DRUJ alignment and osseous congruity, as well as identifying associated fractures. MRI provides complementary information about the soft tissues of the wrist, particularly the TFCC. Injury to the TFCC may contribute to DRUJ instability and may be associated with recurrent instability. In cases of persistent or recurrent instability, surgical treatment may be required to address the associated ligamentous injury [1]. In the present case, neither CT nor MRI was performed because of resource limitations at our healthcare facility.

Mino et al. compared CT and radiographic findings in 15 wrists and found that, among nine wrists that appeared normal on radiographs, four demonstrated DRUJ dislocation on CT. This finding demonstrates the importance of detailed imaging evaluation in such cases [8].

Rapid confirmation of the diagnosis and prompt intervention usually lead to favorable outcomes, as demonstrated in the case series published by Mulford et al., in which closed reduction was performed in 11 patients in the emergency department on the same day as the injury. However, there are also reports of cases that benefited from closed reduction as late as seven weeks after injury [2,9].

Once the diagnosis is established, closed reduction should be performed under local anesthesia, with or without sedation. The maneuver consists of traction applied to the affected wrist, accompanied by dorsal or volar pressure to translate the ulnar head, combined with supination or pronation, depending on the type of dislocation. If joint instability persists after reduction, percutaneous fixation with Kirschner wires may be required. Other fixation options, such as the DRUJ TightRope (suture-button) technique, may also be considered, depending on the degree of instability and the associated soft-tissue injury [10].

## Conclusions

Acute isolated volar dislocation of the DRUJ is a rare injury that may be overlooked during the initial evaluation. Early clinical recognition, careful radiographic assessment, and prompt reduction are important for restoring joint alignment and stability. In this case, closed reduction followed by percutaneous stabilization resulted in satisfactory short-term clinical and radiographic outcomes, with no pain, preserved pronation and supination, and clinical stability of the DRUJ at two months. However, long-term functional recovery and the potential for chronic pain, recurrent instability, or limitations in forearm rotation could not be assessed because the patient was lost to follow-up.
